# The Pathological Society

**Published:** 1896-10-24

**Authors:** 


### The Pathological Society.

On Tuesday evening, October 20th, the Pathological Society celebrated the fiftieth anniversary of its foundation by a conversazione.

The members and guests assembled in large numbers, and after being received by Mr. Butlin, the president, distributed themselves through the various rooms, in which a collection of specimens of historical and pathological interest had been collected together for the occasion.

The pathological specimens exhibited were in many instances the first which had been shown before the society to illustrate the chief advances in morbid anatomy during the past fifty years. These had been collected from many museums, and a catalogue, which had been drawn up by Dr. Newton Pitt and Mr. J. H. Targett, together with Dr. Cayley, Mr. D'Arcy Power, and Mr. S. G. Shattock, was presented to the visitors, setting forth the earliest records made to the society of the chief pathological lesions of more general interest. No one who looked over the specimens exhibited, and bore in mind that nearly all of them marked the date of the acceptance by the profession of new knowledge, could fail to be struck with the large extent to which our present pathological faith has been built up within this generation.

The President, in addressing the meeting, said that there still remained to the society five of its original members, who had met fifty years before at its first meeting, which curiously took place, then as now, on Tuesday, October 20th, the late Dr. C. J. B. Williams, the first president, being in the chair. From the beginning the society had been practical, and had devoted itself to morbid anatomy, eschewing all abstract discussions, and limiting its work to the study of specimens and preparations. Even before the origin of the society a great deal of pathological work was being done in London—King, Walshe, Todd, and Paget being all engaged in teaching. Still, there were only six medical schools at that time which professed to give any teaching in pathology, and that was probably much mingled with other subjects. Although, however, there were then some excellent pathologists, the study of the subject had not taken hold among the students, and among the teachers at some of the schools it was held in a certain contempt. Mr. Butlin related how he himself, years later, had been warned not to have too much to do with the Pathological Society if he wished to rank as a surgeon. Fifty years, however, had made a great difference. Instead of a few books on the general subject, treatises abounded devoted to its every branch; there were journals which dealt exclusively with pathological questions, some of which even confined themselves to particular aspects of the science; pathological laboratories had been formed in connection with most of the schools; a good knowledge of pathology had come to be necessary to everyone who aimed at the position of a consultant; and it might even be said that the tendency was now for the practitioner to rely almost unduly on the pathologist and bacteriologist in the diagnosis of disease. He could not claim for the Pathological Society any one pathological discovery or any new pathological fact; yet its influence in encouraging pathological work had been most important, and its "Transactions," containing upwards of 7,000 separate communications, formed the most valuable record of morbid anatomy which the world possessed, presenting a fair transcript of the trend taken by pathology from time to time during the last fifty years. Comparing the present with the past, however, he could not but feel that the society had reached and had passed a climax in its prosperity. The life of a society had some resemblance to that of a man, and whether he looked at the character of the meetings, or the bulk of the transactions, or the number of applications for membership, he could not help questioning whether the society were not tending towards that slow atrophy by which old people were sometimes affected. This was, no doubt, partly due to the increasing specialisation which was taking place in pathology, as in every other science, as the result of which the communications became interesting to a much smaller circle than formerly. In a way, this indicated the high character of the work done; but those who knew how the Pathological Society had for fifty years served as a point of reunion by aid of which men who had become severed from hospitals maintained their touch with pathological work, would, with him, be sorry to find such men ceasing to take an interest in its meetings in consequence of their specialisation in the direction of bacteriological and chemical pathology—a specialisation which, he feared, was likely only to attract small audiences. A vote of thanks to the President for his address was proposed by Sir Richard Quain, and seconded by Dr. Wilks.

